# Lung Transplant Recipients Immunogenicity after Heterologous ChAdOx1 nCoV-19—BNT162b2 mRNA Vaccination

**DOI:** 10.3390/v14071470

**Published:** 2022-07-02

**Authors:** Emilie Catry, Julien Favresse, Constant Gillot, Jean-Louis Bayart, Damien Frérotte, Michel Dumonceaux, Patrick Evrard, François Mullier, Jonathan Douxfils, François M. Carlier, Mélanie Closset

**Affiliations:** 1 Department of Laboratory Medicine, CHU UCL Namur, Université Catholique de Louvain, 5530 Yvoir, Belgium; emilie.catry@student.uclouvain.be (E.C.); francois.mullier@chuuclnamur.uclouvain.be (F.M.); 2 Department of Laboratory Medicine, Clinique St-Luc Bouge, 5004 Bouge, Belgium; julien.favresse@slbo.be; 3 Department of Pharmacy, Namur Research Institute for Life Sciences, University of Namur, 5000 Namur, Belgium; constant.gillot@unamur.be (C.G.); jonathan.douxfils@unamur.be (J.D.); 4 Department of Laboratory Medicine, Clinique Saint-Pierre, 1340 Ottignies, Belgium; jean-louis.bayart@cspo.be; 5 Lung Transplant Centre, CHU UCL Namur, Université Catholique de Louvain, 5530 Yvoir, Belgium; damien.frerotte@chuuclnamur.uclouvain.be (D.F.); michel.dumonceaux@chuuclnamur.uclouvain.be (M.D.); patrick.evrard@chuuclnamur.uclouvain.be (P.E.); francois.carlier@chuuclnamur.uclouvain.be (F.M.C.); 6 Department of Pneumonology, CHU UCL Namur, Université Catholique de Louvain, 5530 Yvoir, Belgium; 7 Department of Intensive Care Medicine, CHU UCL Namur, Université Catholique de Louvain, 5530 Yvoir, Belgium; 8 Qualiblood s.a., 5000 Namur, Belgium; 9 Pole of Pneumology, ENT, and Dermatology, Institut de Recherche Expérimentale et Clinique (IREC), Université Catholique de Louvain, 1200 Brussels, Belgium

**Keywords:** lung transplant recipients, heterologous vaccination, ChAdOx1 nCoV-19, BNT162b2 mRNA, SARS-CoV-2, COVID-19, vaccination

## Abstract

(1) Background: High immunosuppressive regimen in lung transplant recipients (LTRs) hampers the immune response to vaccination. We prospectively investigated the immunogenicity of heterologous ChAdOx1 nCoV-19-BNT162b2 mRNA vaccination in an LTR cohort. (2) Methods: Forty-nine COVID-19 naïve LTRs received a two-dose regimen ChAdOx1 nCoV-19 vaccine. A subset of 32 patients received a booster dose of BNT162b2 mRNA vaccine 18 weeks after the second dose. (3) Results: Two-doses of ChAdOx1 nCoV-19 induced poor immunogenicity with 7.2% seropositivity at day 180 and low neutralizing capacities. The BNT162b2 mRNA vaccine induced significant increases in IgG titers with means of 197.8 binding antibody units per milliliter (BAU/mL) (95% CI 0–491.4) and neutralizing antibodies, with means of 76.6 AU/mL (95% CI 0–159.6). At day 238, 32.2% of LTRs seroconverted after the booster dose. Seroneutralization capacities against Delta and Omicron variants were found in only 13 and 9 LTRs, respectively. Mycophenolate mofetil and high-dose corticosteroids were associated with a weak serological response. (4) Conclusions: The immunogenicity of a two-dose ChAdOx1 nCoV-19 vaccine regimen was very poor in LTRs, but was significantly enhanced after the booster dose in one-third of LTRs. In immunocompromised individuals, the administration of a fourth dose may be considered to increase the immune response against SARS-CoV-2.

## 1. Introduction

Lung transplant recipients (LTRs) are considered at higher risk of developing severe SARS-CoV-2 pneumonia due to chronic immunosuppressive regimen and underlying comorbid conditions [1,2,3].

Impaired immunogenicity following a two-dose regimen anti-SARS-CoV-2 vaccine has been observed in solid organ transplant recipients (SOTRs), mainly focusing on mRNA-based vaccines [4,5,6,7,8]. However, the immune response has been investigated only in small numbers of LTRs [4,5,6], in whom the vaccination effectiveness is particularly challenging given the concomitant administration of high doses of immunosuppressive agents [3].

Heterologous vaccination (i.e., primary course with two different COVID-19 vaccines or the use of a third booster dose of a different COVID-19 vaccine) has been shown to induce strong immune responses, inducing improved humoral and cellular immune responses in the general population and immunocompromised individuals [8,9,10]. Based on currently available evidence, both the European Medicines Agency and the European Centre for Disease Prevention and Control recommend a heterologous vaccination course against COVID-19, either in the primary course or as a booster [11].

However, data regarding the immunogenicity of a third vaccine dose and heterologous vaccination in LTRs are scarce. In the present study, we aimed at evaluating the immune response to the two-dose regimen of ChAdOx1 nCoV-19 vaccine in a prospective cohort of LTRs up to 6 months after the first administration. In addition, we investigated the 28-day impact of the heterologous third dose (BNT162b2 mRNA vaccine) on the immune response in a subset of LTR participants.

## 2. Materials and Methods

### 2.1. Study Participants

**Figure 1:**
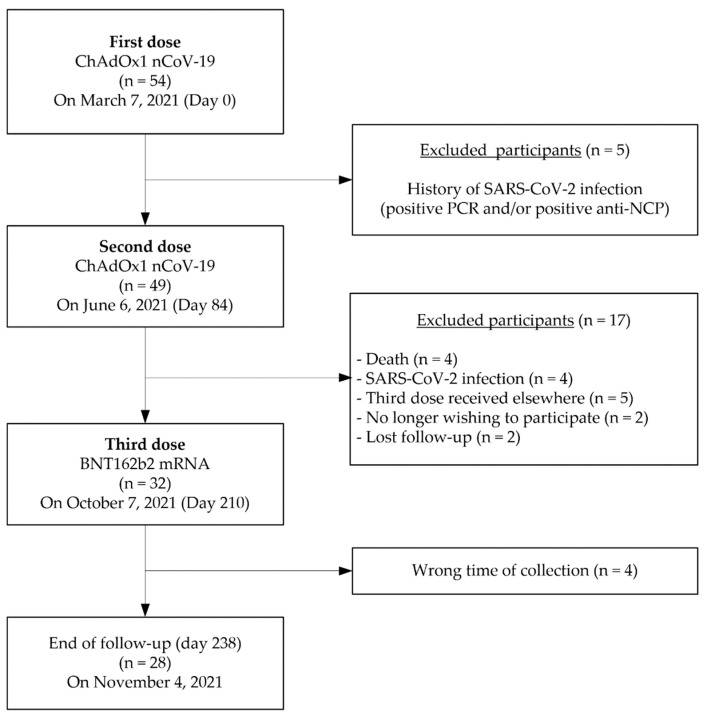
STROBE, STrengthening the Reporting of OBservational studies in Epidemiology.

Fifty-four LTRs were vaccinated against COVID-19 with a two-dose regimen ChAdOx1 nCoV-19 vaccine (AZD1222, AstraZeneca, Cambridge, UK) on 7 March 2021 (day 0). The second dose was administered 12 weeks later, on 6 June 2021 (day 84). The third heterologous dose of BNT162b2 mRNA COVID-19 vaccine (Comirnaty^®^, Pfizer/BioNtech, Mainz, Germany) was administered 18 weeks after the second dose, on 7 October 2021 (at day 210). Figure 1 summarizes the STROBE flow-chart of the study cohort whose main demographic characteristics are recapitulated in Table 1.

Five LTRs with previous SARS-CoV-2 infection (documented by a positive PCR and/or positive antibodies against the nucleocapsid (NCP)) were excluded. Forty-nine LTRs without previous SARS-CoV-2 infection were considered COVID-19-naïve before the study and enrolled in the present ongoing prospective and non-interventional clinical trial, approved by an ethical commission (extension of the study protocol of the CRO-VAX-HCP study, EudraCT registration number: 2020-006149-21, CE Mont-Godinne 01/2021) [12]. Among them, 32 received the third booster dose at day 210, and 28 pursued the investigation up to day 238.

All vaccinated and enrolled LTRs provided written informed consent prior to data and specimen collection. Demographic and clinical data were retrieved from their electronic medical record at baseline.

### 2.2. Samples Collection

Serum samples were collected before the administration of the first vaccine dose (day 0; maximum 48 h before vaccination) (n = 49) and 28 (n = 47), 84 (n = 49), 112 (n = 44), 180 (n = 42), 210 (n = 32) and 238 (n = 28) days after the first dose.

A total of 291 sera were aliquoted and stored at −80 °C until analysis. Sera at days 84 and 210 were collected prior to the administration of the second ChadOx1 nCov-19 and booster BNT162b2 dose, respectively.

### 2.3. Analytical Procedures for Evaluation of Immunogenicity

Total antibodies against the SARS-CoV-2 Nucleocapsid (NCP) (Elecsys^®^ Anti-SARS-CoV-2 NCP qualitative ECLIA, Roche Diagnostics, Machelen, Belgium) were measured to document a previous infectious episode. According to the manufacturer’s instructions, the positivity threshold cutoff was set to 1.

Anti-SARS-CoV-2 responses were assessed and performed on a VITROS^®^ 5600 integrated system (Ortho Clinical Diagnostics, New Jersey, USA). All samples were analyzed using anti-SARS-CoV-2 IgG quantitative immunoassays (Ortho Clinical Diagnostics) against the trimeric spike S1 protein. The results are expressed as binding antibody units per milliliter (BAU/mL), and results < 2 and >4000 BAU/mL (lower and upper limits of quantification) were rounded to 2 and 4000 BAU/mL for statistical analyses. The positivity cut-off was set to ≥17.8 BAU/mL as reported by the manufacturer. 

The neutralizing capacity of antibodies (NAbs) was estimated as previously described [13]. Briefly, NAbs inhibiting the binding of the receptor-binding domain of the surface spike to the human angiotensin-converting enzyme 2 receptor were measured by performing a SARS-CoV-2 surrogate virus neutralization test (sVNT) using the iFlash-2019 nCoV NAbs assay (YHLO Biotech Co., Shenzhen, China). The positivity cut-off was set to ≥10 AU/mL according to the manufacturer’s instructions.

All serum samples (n = 291) were assessed using a pseudovirus neutralization test (pVNT) for wild-type SARS-CoV-2 (referred to as SARS-CoV-2 spike protein carrying the original D614 genotype). Samples with positive NAbs for the wild-type virus (n = 45) were also assessed for the Delta and the Omicron variants. A sample was considered negative if the half maximal inhibitory concentration (IC50) value of that sample was less than 1:20 dilution [14,15].

### 2.4. Statistical Analyses

Quantitative data regarding the serological response are expressed in log_10_ (geometric mean ± 95% confidence interval (CI)) and descriptive statistics were used to analyze the demographic and clinical data. Statistical significance across longitudinal collection time-points was assessed on raw data by the Kruskal–Wallis test followed by Dunn’s multiple comparison tests. The Mann–Whitney test was performed to evaluate the impact of immunosuppressive regimen on anti-SARS-CoV-2 IgG titers. A chi-square test or a Fisher’s exact test was used to determine if there was a significant relationship between two variables. A *p*-value < 0.05 was considered statistically significant. Statistical analyses were performed using GraphPad Prism (v 9.2.0 for Windows, GraphPad Software, San Diego, CA, USA).

## 3. Results

### 3.1. Demographic and Clinical Characteristics of Lung Transplant Recipients

The baseline characteristics of the study population are summarized in Table 1. Among the 49 included participants, 24 (48.9%) were women (median age = 63, interquartile range (IQR) 58–66) and 25 (51.1%) were men (median age = 67, IQR 62–68). The initial respiratory diseases that led to lung transplant were chronic obstructive pulmonary disease in 34 (69.4%) LTRs and pulmonary fibrosis in 7 (14.3%) LTRs, while rarer respiratory diseases (cystic fibrosis, lymphangioleiomyomatosis, chronic lung allograft dysfunction and pulmonary hypertension) accounted for 16.3% of the total. The median time between transplantation and the first dose of vaccine was 3.8 years (IQR 1.8–6.1). The immunosuppressive regimen included corticosteroids and calcineurin inhibitors (tacrolimus or cyclosporine) in all patients (n = 49) and the addition in most patients an antimetabolite (mycophenolate mofetil [n = 25] or azathioprine [n = 11]) or everolimus (n = 8) (Figure 1 and Table 1).

### 3.2. Seroconversion and Neutralizing Capacity in Vaccinated LTRs

**Figure 2:**
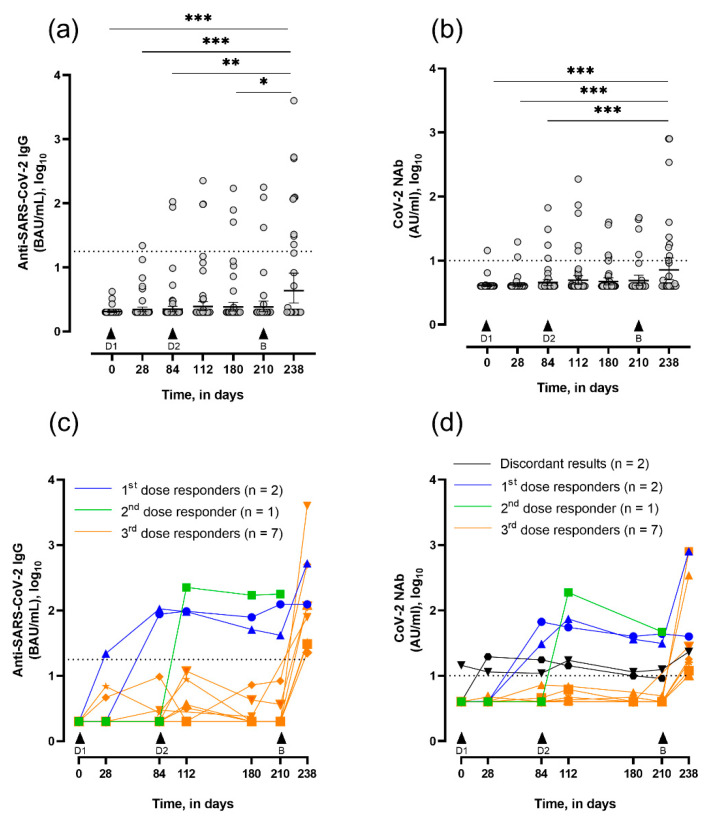
Evolution of serum SARS-CoV-2 IgG titers and neutralizing capacity in vaccinated LTRs, assessed at days 0 (n = 49), 28 (n = 47), 84 (n = 49), 112 (n = 44), 180 (n = 42), 210 (n = 32) and 238 (n = 28). Data are expressed in log_10_. (**a**) Quantitative titers of anti-SARS-CoV-2 IgG at consecutive timepoints (geometric mean in BAU/mL ± 95% CI). * *p* <0.05, ** *p* <0.01, and *** *p* <0.001 by the Kruskal–Wallis test followed by a post–hoc Dunn’s tests. (**b**) Quantitative titers of neutralizing antibodies (NAbs) at consecutive time-points (geometric mean in AU/mL ± 95% CI). *** *p* <0.001 by the Kruskal–Wallis test followed by a post hoc Dunn’s tests. (**c**) Evolution of anti-SARS-CoV-2 IgG titers in seropositive LTRs with first dose responders represented in blue (n = 2), second dose responder in green (n = 1), and booster dose responders in orange (n = 7). (**d**) Evolution of NAbs titers in seropositive LTRs with first dose responders represented in blue (n = 2), second dose responder in green (n = 1), and booster dose responders in orange (n = 7). Patients with discordant results between IgG and NAbs are represented in black (n = 2). Dotted black lines represent the positivity cut-offs for IgG measurement (≥17.8 BAU/mL, i.e., 1.25 in log_10_) and for NAbs measurement (≥10 AU/mL, i.e., 1 in log_10_). D1 and D2, respectively, are the first and second dose of ChAdOx1; B is the booster dose, BNT162b2.

Regarding anti-SARS-CoV-2 IgG titers, no significant modification was observed regarding anti-SARS-CoV-2 IgG titers after two doses of ChadOx1 nCov-19 vaccine in LTRs (i.e., from day 0 to 180) (Figure 2a). At day 180, the serological response rate was 7.2% (Table 2). Two LTRs (4.0%) were considered responders after the first dose, with IgG titers of 87.6 and 106 BAU/mL at day 84, separately (Figure 2c, blue lines). Of the 42 seronegative LTRs, 1 seroconverted after the second dose (IgG titer = 225 BAU/mL) at day 112 (Figure 2c, green line). A subset of participants (n = 32) received the third dose (BNT162b2) at day 210. Twenty-eight days later, the mean anti-SARS-CoV-2 IgG titer was significantly increased compared with all previous collection points. At day 238, the serological response rate was 32.2%, with a mean IgG titer of 608.4 BAU/mL (95% CI: 380.6–1597) in seropositive LTRs (Figure 2c). Seven LTRs were considered booster responders, as they seroconverted after the heterologous vaccine (day 238; Figure 2c, orange lines). LTRs who seroconverted after the two-dose regimen ChadOx1 nCov-19 vaccine (n = 3) kept positive anti-SARS-CoV-2 IgG titers. In addition, the IgG titer increased 12.5-fold after the booster dose to the first one (Figure 2c, blue line with triangle symbol), while the IgG titer to the second one was not influenced by the booster dose (Figure 2c, blue line with circle symbol). The patient who seroconverted after the second dose was not able to provide a sample at the right collection time (Figure 2b, green line). For a small number of participants (n = 4), samples were not collected in due time; thus, immunological analysis was not included.

For sVNT NAbs titers, no significant difference was observed after the two-dose regimen of ChAdOx1 nCoV-19 vaccine in LTRs (i.e., from day 0 to 180). Before vaccination (day 0), one LTR presented positive NAbs titers (14.4 AU/mL), while negative having anti-nucleocapsid titers. This was considered a false-seropositive patient, probably due to interference because NAbs titers did not progress throughout the follow-up. At day 112, five LTRs (11.4%) displayed detectable NAbs titers, with discordant results for two LTRs, which had negative anti-SARS-CoV-2 IgG titers (Figure 2d, black lines and Table 2). None of them had developed previous SARS-CoV-2 infection, as witnessed by negative anti-nucleocapsid titers at day 0 and NAbs titers remaining close to the negativity cut-off with 11.4 and 12.2 AU/mL, respectively. The third dose led to a significant increase in mean NAbs titers at day 238 compared with primary vaccination course time points (days 0, 28, and 84), and increased by 9.5-fold between day 210 and 238. At day 238, nine LTRs (32.2%) displayed positive NAbs titers with a mean of 229 AU/mL (95% CI 0–490.8) (Figure 2d). At day 238, the two IgG-seropositive LTRs after the primary vaccination course showed similar trends in NAbs titers compared to their IgG titer evolution (Figure 2c,d, blue lines).

**Figure 3:**
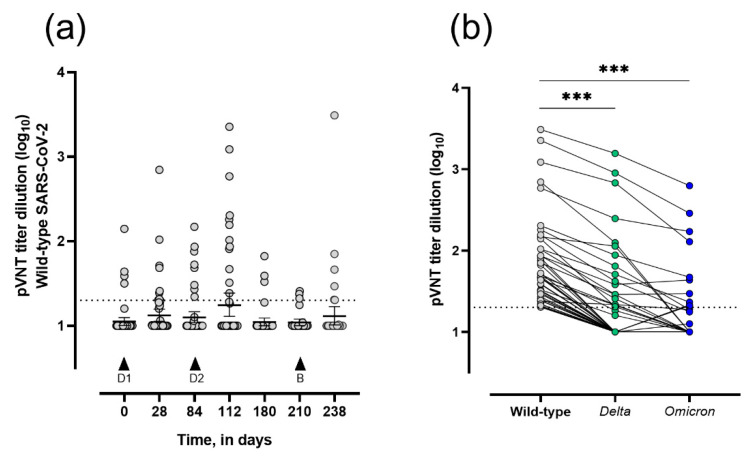
Evolution of wild-type- and SARS-CoV-2 variant-neutralizing capacities in vaccinated-LTRs. Data are expressed in log_10_. (**a**) Dilution titers of anti-SARS-CoV-2 IgG at day 0 (n = 49), 28 (n = 47), 84 (n = 49), 112 (n = 44), 180 (n = 42), 210 (n = 32), and 238 (n = 28), assessed by pseudovirus neutralization test (pVNT) (geometric mean ± 95% CI). Statistical analysis by Kruskal–Wallis test followed by a post hoc Dunn’s test provided non-significant results. (**b**) Paired Delta and Omicron SARS-CoV-2 neutralizing capacities of wild-type-positive samples (n = 45) assessed by pVNT (geometric mean ± 95% CI). Dotted black lines represent the positivity cut-offs for neutralization (>20 dilution titer, i.e., 1.3 in log_10_). *** *p* < 0.001 wild-type vs. Delta and Omicron by the Kruskal–Wallis test followed by a post hoc Dunn’s test. D1 and D2 correspond to the first and second doses of ChAdOx1, respectively; B corresponds to the booster dose, BNT162b2.

The results of the pseudovirus neutralization test showed that four patients (8.1%) already presented positive wild-type SARS-CoV-2 neutralizing titers with a mean dilution titer of 63.5 (95% CI 18–145) before the first vaccine dose. At day 84, 18.4% of LTRs had positive wild-type SARS-CoV-2 neutralizing titers with a mean dilution titer of 56.8 (95% CI 25.0–88.5). After two doses of the ChAdOx1 nCoV-19 vaccine (day 112), 27.3% of LTRs showed positive wild-type SARS-CoV-2 neutralizing titers with a mean dilution titer of 415.7 (95% CI 13.6–845.0). After the heterologous dose (day 238), 21.4% of LTRs had positive wild-type SARS-CoV-2 neutralizing titers with a mean dilution titer of 110 (95% CI 0–305.6) (Figure 3a). The samples that were wild-type-neutralizing positive (n = 45 issued from 24 different patients) were tested for seroneutralization against the Delta and Omicron variants. Of the 45 samples, 18 (40.0%) demonstrated positive Delta-neutralizing titers (from 13 different LTRs) while 10 (22.2%) had positive Omicron-neutralizing titers (distributed among 9 LTRs) (Figure 3b). Among them, two LTRs presented discordant results between Delta- (negative titers) and Omicron-neutralizing capacities (positive titers). Only 25.0% (6/24) of the LTRs with positive wild-type-neutralizing titers were IgG-seropositive at one or more collection time points. Moreover, 15.4% (2/13) and 11.1% (1/9) of LTRs had Delta- and Omicron-neutralizing titers associated with positive IgG titers for at least one collection time point, respectively.

Table 3 reports the rate of seropositive LTRs with neutralization capacities or complete non-responders after the heterologous ChAdOx1 nCoV-19–BNT162b2 mRNA vaccine according to demographic and clinical characteristics of the participants.

## 4. Discussion

This study highlights that the two-dose regimen of ChAdOx1 nCoV-19 vaccine induces poor immunogenicity in LTRs, as witnessed by the low neutralizing capacities against wild-type and specific SARS-CoV-2 variants. Indeed, 92.8% of the included LTRs failed to mount detectable antibody response following this vaccination regimen. A third heterologous dose with the BNT162b2 vaccine, administered in a subset of LTRs, elicited higher rates of both seroconversion and positive neutralizing antibodies against SARS-CoV-2, observed in 32.2% of triple-vaccinated LTRs. 

Our findings are consistent with previously published data demonstrating weak immune responses after vectored or mRNA vaccines [5,7,16], contrasting the excellent immunogenicity rates found in the general population [12,13,15,17,18,19,20]. 

The immunosuppressive regimen appears to be a key player in preventing the development of immune response. Among SOTRs, LTRs are distinguished by a heavier immunosuppressive regimen, potentially explaining the discrepant response to vaccination observed in our study population compared with other studies. For instance, Schmidt et al. reported 35.3% seropositivity in SOTRs after completion of the two-dose ChAdOx1 nCoV-19 vaccine versus 7.2% of LTRs in our study [8]. Our data nevertheless corroborate previous study results [7,21,22], as only LTRs treated with a low daily dose of corticosteroids reached seropositivity with neutralization capacities (for the wild-type, Delta, and Omicron variants), and immunosuppressive regimens including either mycophenolate mofetil or high-dose corticosteroids were associated with poorer vaccine-induced immune response. However, the threshold for effective antibody protection following SARS-CoV-2 vaccination remains undetermined.

Facing the weak immune response after the completion of a primary vaccination course with vectored or mRNA vaccine in SOTRs, booster doses (third and even a fourth) have been proposed by national and international competent authorities [23,24] to reach and maintain higher immunogenicity levels. Previous studies reported up to 50% seropositivity 4 weeks after a third booster dose in distinct SOTR cohorts [25,26,27,28]. The seropositivity rate and NAbs titers increased more than four-fold following the booster dose in our specific patient cohort compared with the two-dose regimen of ChAdOx1 nCoV-19 vaccine. While the neutralization capacity of antibodies is increasingly considered a predictor of vaccination efficacy [29,30], both seroneutralization techniques, namely sVNT and pVNT, are poorly correlated with each other and with quantitative anti-SARS-CoV-2 immunoassays [13,15,18,31].

Our study has several limitations. Firstly, the national vaccination strategy negatively impacted the recruitment of a two-dose ChAdOx1 nCoV-19 vaccinated control group, although information about immunogenicity after a two-dose ChAdOx1 nCoV-19 regimen in the general population can be found in several previous studies [9,10,17,32,33,34,35,36]. Secondly, our study design prevented us from comparing the effectiveness of a heterologous versus homologous booster dose in LTRs. Thirdly, we did not investigate the cellular immune response, while it has been shown that heterologous vaccination leads to a stronger CD4+ T cells response in the general population as well as in SOTRs [8,9], and vaccine-induced T cell responses are of similar magnitude to those seen after natural infection in immunocompetent individuals [37]. In a recent Letter to the Editor from Havlin et al., emergence of cellular response was reportedly detected in 47% of LTRs (n = 15) after the third vaccine dose [38]. High-affinity NAbs are closely regulated by T follicular helper cells, which are essential for the control of viral infections and vaccine responses by mediating the interaction between T and B cells [39,40,41].

## 5. Conclusions

We showed that a two-dose regimen of ChAdOx1 nCoV-19 vaccine provides poor immunogenic effects on humoral response in LTRs. Our data, specifically collected in LTRs, indicated that a third heterologous dose of BNT162b2 (booster) elicited higher titers of IgG and NAbs after homologous two-dose ChAdOx1 nCoV-19 vaccine. Nevertheless, the rate of three-dose non-responders remained substantial at day 238 with 67.8% of seronegative LTRs in our study population and was even more important regarding Omicron SARS-CoV-2 neutralizing capacities. These results support the strategy of administering a fourth SARS-CoV-2 vaccine dose in immunocompromised patients, a decision that was implemented in January 2022 by the Superior Health Council of Belgium.

## Figures and Tables

**Table 1:** Demographic and clinical characteristics of lung transplant recipients (LTRs).

Participant Characteristics	LTRs (n = 49)
Age, years (median ± IQR)	64 (60.5–68)
Female sex, n (%)	24 (48.9)
White, n (%)	47 (95.9)
Non-white, n (%)	2 (4.1)
Initial respiratory disease, n (%)	
Chronic obstructive pulmonary disease	34 (69.4)
Pulmonary fibrosis	7 (14.3)
Other	8 (16.3)
Types of lung transplantation	
Bi-pulmonary transplantation	47 (95.9)
Mono-pulmonary transplantation	2 (4.1)
Years since transplant, years (median ± IQR)	3.8 (1.8–6.1)
Immunosuppressive regimen	
Corticosteroids (methylprednisolone), n (%)	
2–6 mg/day	41 (83.7)
≥8 mg/day	8 (16.3)
Calcineurin inhibitors, n (%)	
Tacrolimus	43 (87.7)
Cyclosporine	6 (12.2)
Others, n (%)	
Mycophenolate mofetil	25 (51.0)
Azathioprine	11 (22.4)
Everolimus	8 (16.3)
Type of vaccines, n (%)	
Two-dose ChAdOx1 nCoV-19 vaccine	49 (100)
BNT162b2 mRNA COVID-19 vaccine (3rd dose)	32 (65.3)

IQR, interquartile range.

**Table 2:** Anti-SARS-CoV-2 IgG and NAbs titers, and proportion of seropositive LTRs at each collection time point.

	LTR Participants				
Day of Collection	Anti-SARS-CoV-2 IgG (BAU/mL)	IgG-seropositive LTRs, n (%)	NAbs (AU/mL)	NAbs-seropositive LTRs, n (%)	IgG and/or NAbs Seropositive LTRs, n (%)
Day 0 (n = 49)	2.1 (2.0–2.2)	0 (0.0)	4.3 (3.8–4.7)	1 (2.0)	1 (2.0)
Day 28 (n = 47)	2.7 (1.9–3.8)	1 (2.1)	4.2 (3.9–4.5)	2 (4.2)	3 (6.4)
Day 84 (n = 49)	6.1 (0.7–11.6)	2 (4.0)	6.2 (3.3–9.0)	4 (8.2)	4 (8.2)
Day 112 (n = 44)	12.1 (0.4–23.8)	3 (6.8)	11.7 (2.3–21.0)	5 (11.4)	5 (11.4)
Day 180 (n = 42) ^a^	9.7 (0.6–18.7)	3 (7.2)	6.3 (3.8–8.8)	4 (10.0)	5 (11.9)
Day 210 (n = 32) ^b^	12.8 (0.0–26.4)	3 (9.3)	8.0 (3.7–12.2)	4 (12.9)	4 (12.5)
Day 238 (n = 28)	197.8 (0.0–491.4) *	9 (32.2)	76.6 (0–159.6) ^#^	9 (32.2)	9 (32.2)

IgG titers and neutralizing antibodies (NAbs) titers are expressed as mean ± 95% CI. * *p* < 0.05 for comparison between day 238 vs all timepoints by the Kruskal–Wallis test followed by a post hoc Dunn’s test. ^#^ *p* < 0.01 for comparison between day 238 and days 0, 28, and 84 by the Kruskal–Wallis test followed by a post hoc Dunn’s test. ^a^ Two missing values for NAbs measurement due to insufficient serum volume. ^b^ One missing value for NAbs measurement due to insufficient serum volume.

**Table 3:** Demographic and clinical characteristics of LTRs with stratification by antibody and neutralization responses after vaccination scheme completion. Seropositive LTRs with neutralization capacities assessed by sVNT were evaluated at day 238 (n = 9) as well as non-responders (n = 19). Seroneutralization against wild-type SARS-CoV-2 and the Delta and Omicron variants was recorded for LTRs with positive titers for least at one collection time point.

	LTRs, (n)					
	Seropositive with Neutralization Capacities (sVNT), (9) (%)	Seronegative, (19) (%)	*p*-Value	With WT Neutralization Capacities, (24) (%)	With Delta Neutralization Capacities, (13) (%)	With Omicron Neutralization Capacities, (9) (%)
Age, years						
18–39	0 (0.0)	1 (5.3)	ns	1 (4.2)	0 (0.0)	1 (11.1)
40–59	2 (22.2)	2 (10.5)		3 (12.5)	1 (7.7)	1 (11.1)
≥60	7 (77.8)	16 (84.2)		20 (83.3)	12 (92.3)	7 (77.8)
Sex						
Female	6 (66.7)	6 (31.6)	ns	11 (45.8)	5 (38.5)	4 (44.4)
Male	3 (33.3)	13 (68.4)		13 (54.2)	8 (61.5)	5 (55.6)
Time since transplant, years						
<1	0 (0.0)	4 (21.0)	ns	3 (12.5)	1 (7.7)	1 (11.1)
>1	9 (100)	15 (79.0)		21 (87.5)	12 (92.3)	8 (88.9)
Initial respiratory disease						
COPD	8 (88.9)	13 (68.4)	ns	20 (83.4)	11 (84.6)	6 (66.7)
Pulmonary fibrosis	0 (0.0)	3 (15.8)		2 (8.3)	1 (7.7)	1 (11.1)
Other	1 (11.1)	3 (15.8)		2 (8.3)	1 (7.7)	2 (22.2)
Corticosteroids						
2–6 mg/day	9 (100)	15 (79.0)	ns	23 (95.8)	13 (100)	9 (100)
≥8 mg/day	0 (0.0)	4 (21.0)		1 (4.2)	0 (0.0)	0 (0.0)

COPD, chronic obstructive pulmonary disease. ns, non-significant.

## Data Availability

The data presented in this study are available on request from the corresponding author. The data are not publicly available according to ethical committee decision on the conduct of this study.

## References

[1] Messika J., Eloy P., Roux A., Hirschi S., Nieves A., Le Pavec J., Senechal A., Saint Raymond C., Carlier N., Demant X. (2021). COVID-19 in Lung Transplant Recipients. Transplantation.

[2] Myers C.N., Scott J.H., Criner G.J., Cordova F.C., Mamary A.J., Marchetti N., Shenoy K.V., Galli J.A., Mulhall P.D., Brown J.C. (2020). COVID-19 in lung transplant recipients. Transpl. Infect. Dis..

[3] Scharringa S., Hoffman T., van Kessel D.A., Rijkers G.T. (2021). Vaccination and their importance for lung transplant recipients in a COVID-19 world. Expert Rev. Clin. Pharm..

[4] Hallett A.M., Greenberg R.S., Boyarsky B.J., Shah P.D., Ou M.T., Teles A.T., Krach M.R., Lopez J.I., Werbel W.A., Avery R.K. (2021). SARS-CoV-2 messenger RNA vaccine antibody response and reactogenicity in heart and lung transplant recipients. J. Heart Lung Transpl..

[5] Havlin J., Svorcova M., Dvorackova E., Lastovicka J., Lischke R., Kalina T., Hubacek P. (2021). Immunogenicity of BNT162b2 mRNA COVID-19 vaccine and SARS-CoV-2 infection in lung transplant recipients. J. Heart Lung Transpl..

[6] Narasimhan M., Mahimainathan L., Clark A.E., Usmani A., Cao J., Araj E., Torres F., Sarode R., Kaza V., Lacelle C. (2021). Serological Response in Lung Transplant Recipients after Two Doses of SARS-CoV-2 mRNA Vaccines. Vaccines.

[7] Peled Y., Ram E., Lavee J., Sternik L., Segev A., Wieder-Finesod A., Mandelboim M., Indenbaum V., Levy I., Raanani E. (2021). BNT162b2 vaccination in heart transplant recipients: Clinical experience and antibody response. J. Heart Lung Transpl..

[8] Schmidt T., Klemis V., Schub D., Schneitler S., Reichert M.C., Wilkens H., Sester U., Sester M., Mihm J. (2021). Cellular immunity predominates over humoral immunity after homologous and heterologous mRNA and vector-based COVID-19 vaccine regimens in solid organ transplant recipients. Am. J. Transpl..

[9] Pozzetto B., Legros V., Djebali S., Barateau V., Guibert N., Villard M., Peyrot L., Allatif O., Fassier J.B., Massardier-Pilonchery A. (2021). Immunogenicity and efficacy of heterologous ChAdOx1-BNT162b2 vaccination. Nature.

[10] Liu X., Shaw R.H., Stuart A.S.V., Greenland M., Aley P.K., Andrews N.J., Cameron J.C., Charlton S., Clutterbuck E.A., Collins A.M. (2021). Safety and immunogenicity of heterologous versus homologous prime-boost schedules with an adenoviral vectored and mRNA COVID-19 vaccine (Com-COV): A single-blind, randomised, non-inferiority trial. Lancet.

[11] European Medicines Agency Heterologous Primary and Booster COVID-19 Vaccination, Evidence Based Regulatory Considerations (EMA/349565/2021). Issued on 13 December 2021. https://www.ema.europa.eu/en/documents/report/heterologous-primary-booster-covid-19-vaccination-evidence-based-regulatory-considerations_en.pdf.

[12] Favresse J., Bayart J.L., Mullier F., Dogne J.M., Closset M., Douxfils J. (2021). Early antibody response in health-care professionals after two doses of SARS-CoV-2 mRNA vaccine (BNT162b2). Clin. Microbiol. Infect..

[13] Favresse J., Gillot C., Di Chiaro L., Eucher C., Elsen M., Van Eeckhoudt S., David C., Morimont L., Dogne J.M., Douxfils J. (2021). Neutralizing Antibodies in COVID-19 Patients and Vaccine Recipients after Two Doses of BNT162b2. Viruses.

[14] Gillot C., Favresse J., Maloteau V., Dogne J.M., Douxfils J. (2022). Identification of SARS-CoV-2 neutralizing antibody with pseudotyped virus-based test on HEK293T hACE2 cells. Bio-Protocol.

[15] Gillot C., Favresse J., Maloteau V., Dogne J.M., Douxfils J. (2021). Dynamics of Neutralizing Antibody Responses Following Natural SARS-CoV-2 Infection and Correlation with Commercial Serologic Tests. A Reappraisal and Indirect Comparison with Vaccinated Subjects. Viruses.

[16] Boyarsky B.J., Werbel W.A., Avery R.K., Tobian A.A.R., Massie A.B., Segev D.L., Garonzik-Wang J.M. (2021). Antibody Response to 2-Dose SARS-CoV-2 mRNA Vaccine Series in Solid Organ Transplant Recipients. JAMA.

[17] Voysey M., Clemens S.A.C., Madhi S.A., Weckx L.Y., Folegatti P.M., Aley P.K., Angus B., Baillie V.L., Barnabas S.L., Bhorat Q.E. (2021). Safety and efficacy of the ChAdOx1 nCoV-19 vaccine (AZD1222) against SARS-CoV-2: An interim analysis of four randomised controlled trials in Brazil, South Africa, and the UK. Lancet.

[18] Favresse J., Gillot C., Douxfils J. (2021). Reply to Schulte-Pelkum, J. Comment on “Favresse et al. Persistence of Anti-SARS-CoV-2 Antibodies Depends on the Analytical Kit: A Report for Up to 10 Months after Infection. *Microorganisms* 2021, 9, 556”. Microorganisms.

[19] Bayart J.L., Morimont L., Closset M., Wieers G., Roy T., Gerin V., Elsen M., Eucher C., Van Eeckhoudt S., Ausselet N. (2021). Confounding Factors Influencing the Kinetics and Magnitude of Serological Response Following Administration of BNT162b2. Microorganisms.

[20] Favresse J., Bayart J.L., Mullier F., Elsen M., Eucher C., Van Eeckhoudt S., Roy T., Wieers G., Laurent C., Dogne J.M. (2021). Antibody titres decline 3-month post-vaccination with BNT162b2. Emerg. Microbes Infect..

[21] Peled Y., Ram E., Lavee J., Segev A., Matezki S., Wieder-Finesod A., Halperin R., Mandelboim M., Indenbaum V., Levy I. (2021). Third dose of the BNT162b2 vaccine in heart transplant recipients: Immunogenicity and clinical experience. J. Heart Lung Transpl..

[22] Aslam S., Danziger-Isakov L., Mehra M.R. (2021). COVID-19 vaccination immune paresis in heart and lung transplantation. J. Heart Lung Transpl..

[23] Centers for Disease Control and Prevention (CDC) Interim Clinical Considerations for Use of COVID-19 Vaccines Currently Approved or Authorized in the United States. Issued on 28 December 2021. https://www.cdc.gov/vaccines/covid-19/downloads/summary-interim-clinical-considerations.pdf.

[24] Centre Fédéral d’Expertise des Soins de Santé (KCE) Rapid Review of the Evidence on a COVID-19 Booster Dose after a Primary Vaccination Schedule, Report for the TASK Force Vaccination. Issued on 17 August 2021. https://kce.fgov.be/sites/default/files/atoms/files/Third%20Covid-19%20vaccination_Report_DUTCH.pdf.

[25] Del Bello A., Abravanel F., Marion O., Couat C., Esposito L., Lavayssiere L., Izopet J., Kamar N. (2021). Efficiency of a boost with a third dose of anti-SARS-CoV-2 messenger RNA-based vaccines in solid organ transplant recipients. Am. J. Transpl..

[26] Werbel W.A., Boyarsky B.J., Ou M.T., Massie A.B., Tobian A.A.R., Garonzik-Wang J.M., Segev D.L. (2021). Safety and Immunogenicity of a Third Dose of SARS-CoV-2 Vaccine in Solid Organ Transplant Recipients: A Case Series. Ann. Intern. Med..

[27] Benotmane I., Gautier G., Perrin P., Olagne J., Cognard N., Fafi-Kremer S., Caillard S. (2021). Antibody Response After a Third Dose of the mRNA-1273 SARS-CoV-2 Vaccine in Kidney Transplant Recipients With Minimal Serologic Response to 2 Doses. JAMA.

[28] Massa F., Cremoni M., Gerard A., Grabsi H., Rogier L., Blois M., Couzin C., Hassen N.B., Rouleau M., Barbosa S. (2021). Safety and cross-variant immunogenicity of a three-dose COVID-19 mRNA vaccine regimen in kidney transplant recipients. EBioMedicine.

[29] Khoury D.S., Cromer D., Reynaldi A., Schlub T.E., Wheatley A.K., Juno J.A., Subbarao K., Kent S.J., Triccas J.A., Davenport M.P. (2021). Neutralizing antibody levels are highly predictive of immune protection from symptomatic SARS-CoV-2 infection. Nat. Med..

[30] Bayart J.L., Douxfils J., Gillot C., David C., Mullier F., Elsen M., Eucher C., Van Eeckhoudt S., Roy T., Gerin V. (2021). Waning of IgG, Total and Neutralizing Antibodies 6 Months Post-Vaccination with BNT162b2 in Healthcare Workers. Vaccines.

[31] Favresse J., Eucher C., Elsen M., Gillot C., Van Eeckhoudt S., Dogne J.M., Douxfils J. (2021). Persistence of Anti-SARS-CoV-2 Antibodies Depends on the Analytical Kit: A Report for Up to 10 Months after Infection. Microorganisms.

[32] Barrett J.R., Belij-Rammerstorfer S., Dold C., Ewer K.J., Folegatti P.M., Gilbride C., Halkerston R., Hill J., Jenkin D., Stockdale L. (2021). Phase 1/2 trial of SARS-CoV-2 vaccine ChAdOx1 nCoV-19 with a booster dose induces multifunctional antibody responses. Nat. Med..

[33] Folegatti P.M., Ewer K.J., Aley P.K., Angus B., Becker S., Belij-Rammerstorfer S., Bellamy D., Bibi S., Bittaye M., Clutterbuck E.A. (2020). Safety and immunogenicity of the ChAdOx1 nCoV-19 vaccine against SARS-CoV-2: A preliminary report of a phase 1/2, single-blind, randomised controlled trial. Lancet.

[34] Ramasamy M.N., Minassian A.M., Ewer K.J., Flaxman A.L., Folegatti P.M., Owens D.R., Voysey M., Aley P.K., Angus B., Babbage G. (2021). Safety and immunogenicity of ChAdOx1 nCoV-19 vaccine administered in a prime-boost regimen in young and old adults (COV002): A single-blind, randomised, controlled, phase 2/3 trial. Lancet.

[35] Schmidt T., Klemis V., Schub D., Mihm J., Hielscher F., Marx S., Abu-Omar A., Ziegler L., Guckelmus C., Urschel R. (2021). Immunogenicity and reactogenicity of heterologous ChAdOx1 nCoV-19/mRNA vaccination. Nat. Med..

[36] Voysey M., Costa Clemens S.A., Madhi S.A., Weckx L.Y., Folegatti P.M., Aley P.K., Angus B., Baillie V.L., Barnabas S.L., Bhorat Q.E. (2021). Single-dose administration and the influence of the timing of the booster dose on immunogenicity and efficacy of ChAdOx1 nCoV-19 (AZD1222) vaccine: A pooled analysis of four randomised trials. Lancet.

[37] Moss P. (2022). The T cell immune response against SARS-CoV-2. Nat. Immunol..

[38] Havlin J., Skotnicova A., Dvorackova E., Hubacek P., Svorcova M., Lastovicka J., Sediva A., Kalina T., Lischke R. (2022). Impaired Humoral Response to Third Dose of BNT162b2 mRNA COVID-19 Vaccine Despite Detectable Spike Protein-specific T cells in Lung Transplant Recipients. Transplantation.

[39] Dudreuilh C., Basu S., Scottà C., Dorling A., Lombardi G. (2021). Potential Application of T-Follicular Regulatory Cell Therapy in Transplantation. Front. Immunol..

[40] Yan L., de Leur K., Hendriks R.W., van der Laan L.J.W., Shi Y., Wang L., Baan C.C. (2017). T Follicular Helper Cells As a New Target for Immunosuppressive Therapies. Front. Immunol..

[41] Cui D., Tang Y., Jiang Q., Jiang D., Zhang Y., Lv Y., Xu D., Wu J., Xie J., Wen C. (2021). Follicular Helper T Cells in the Immunopathogenesis of SARS-CoV-2 Infection. Front. Immunol..

